# Microtubule-modulating Agents in the Fight Against Neurodegeneration: Will it ever Work?

**DOI:** 10.2174/1570159X19666211201101020

**Published:** 2022-03-28

**Authors:** Ahmed Soliman, Lidia Bakota, Roland Brandt

**Affiliations:** 1 Department of Neurobiology, Osnabrück University, Osnabrück, Germany;; 2 Center for Cellular Nanoanalytics, Osnabrück University, Osnabrück, Germany;; 3 Institute of Cognitive Science, Osnabrück University, Osnabrück, Germany

**Keywords:** Microtubules, cytoskeleton, neurodegenerative diseases, tauopathies, microtubule-modulating drugs, neuron

## Abstract

The microtubule skeleton plays an essential role in nerve cells as the most important structural determinant of morphology and as a highway for axonal transport processes. Many neurodegenerative diseases are characterized by changes in the structure and organization of microtubules and microtubule-regulating proteins such as the microtubule-associated protein tau, which exhibits characteristic changes in a whole class of diseases collectively referred to as tauopathies. Changes in the dynamics of microtubules appear to occur early under neurodegenerative conditions and are also likely to contribute to age-related dysfunction of neurons. Thus, modulating microtubule dynamics and correcting impaired microtubule stability can be a useful neuroprotective strategy to counteract the disruption of the microtubule system in disease and aging. In this article, we review current microtubule-directed approaches for the treatment of neurodegenerative diseases with microtubules as a drug target, tau as a drug target, and post-translational modifications as potential modifiers of the microtubule system. We discuss limitations of the approaches that can be traced back to the rather unspecific mechanism of action, which causes undesirable side effects in non-neuronal cell types or which are due to the disruption of non-microtubule-related interactions. We also develop some thoughts on how the specificity of the approaches can be improved and what further targets could be used for modulating substances.

## 1 INTRODUCTION

### 1.1 The Neuronal Microtubule Cytoskeleton

The cytoskeleton is the most important intracellular determinant of cell shape. One of the main components of the cytoskeleton are microtubules, long and straight hollow cylinders, which in neurons preferably consist of 13 laterally associated protofilaments [1 Microtubules are inherently polar structures that provide structural stability and form a track for directed motor-protein dependent transport [2 This is particularly important for nerve cells that have a strong functional and morphological polarization into an axonal and a somatodendritic compartment. The compartmentalization of neurons is also reflected in the organization of the microtubule skeleton, which differs in the soma, the dendrites, and the axon [3 In the soma, microtubules emanate from a microtubule-organizing center and are anchored there, similar to the organization of microtubules in most other mammalian cell types. In contrast, microtubules in the dendrite and axon are discontinuous, where they have multiple start and stop sites. The organisation of the microtubule array also differs between axons and dendrites, as microtubules in the axon are uniformly orientated with their plus-end pointing outwards, while in the dendrites, they have a mixed orientation [4 The uniform orientation of the microtubules is of particular importance for axons that crucially depend on efficient transport, especially since in some neurons, the axon can be more than a meter long.

Microtubules are dynamic structures, and their organization and dynamics are important for the development of processes and the maintenance of the structural and functional plasticity of neurons [5, 6 The dynamics of microtubules show strong local variations and are regulated by a large number of associated proteins that modulate the assembly state, the organization, and the stability of the microtubule skeleton in a spatially restricted manner [7 Such associated proteins include microtubule-associated proteins (MAPs), which bind to microtubule polymer on one side, and proteins such as stathmin, which bind to soluble tubulin dimers on the other, thereby shifting the polymerization/depolymerization equilibrium either to the polymer or to the dimer state [8, 9 In addition, plus-end binding proteins (+TIPs) dynamically accumulate at the growing end of microtubules, and microtubule severing enzymes such as katanin and spastin cause MT fragmentation [10, 11 Bioinformatic analyses have suggested that changes in MAPs and tubulin-sequestering proteins in particular are important drivers for the development of increased neuronal complexity during mammalian evolution [12], which indicates the importance of these two protein classes for the development of neurons and the maintenance of their structure.

Paradigmatic examples of MAPs in nerve cells are the neuronal MAPs tau and MAP2, which show a compartment-specific distribution in mature neurons, with tau accumulating in the axon, while MAP2 is mainly present in the somatodendritic compartment [13 Originally it was assumed that MAPs like tau and MAP2 stabilize microtubules in their respective compartments. However, recent data has shown that they have more complex functions; in fact, their primary role may not be to stabilize microtubules since, for example, mice knocked out in tau show only minor phenotypic changes with no apparent change in microtubule stability [14 It is now recognized that MAPs, in addition to microtubules, can have multiple interaction partners and serve as proteins that anchor components of signal cascades and can dynamically connect the microtubule skeleton to other cellular structures such as the neuronal plasma membrane [15-17 It has also been recognized that MAPs are highly dynamic and exhibit kiss-and-hop interaction with microtubules, and their interactions are modulated by phosphorylation and probably other post-translational modifications as well [18, 19

Further complexity arises from the fact that microtubules themselves undergo several post-translational modifications. These modifications include acetylation, tyrosination, glutamylation, and glycylation, just to name a few. These modifications have the potential to create a wide variety of microtubule identities known as the “tubulin code” [20 These modifications can, in turn, affect the interaction with microtubule-binding proteins, including motor proteins, whereby a mutual influence of several actors of the microtubule system arises.

## 2 CHANGES IN THE STRUCTURE AND ORGANIZATION OF MICROTUBULES AND MICROTUBULE- REGULATING PROTEINS DURING AGING AND NEURODEGENERATIVE DISEASES

Given the important role of the microtubule system in the structure, function, and plasticity of neurons, it is not surprising that changes in the organization of microtubules and microtubule-regulating proteins are common with aging and in neurodegenerative diseases. An example is the observation of a significant age-dependent decrease in the microtubule density in neuronal cells of older people and a significant reduction in the number and length of microtubules in pyramidal neurons from the brain of Alzheimer's disease (AD) patients compared to control cases [21 Changes in microtubules, particularly decreased levels of acetylated tubulin, a marker of a more stable microtubule subpopulation, have also been observed in patients with other neurodegenerative diseases such as Huntington’s disease. Table **1** shows some examples of microtubule changes in several neurodegenerative diseases.

Characteristic changes in MAPs during aging and in neurodegenerative diseases such as AD have also been described. For example, an age-dependent reduction in MAP2 in the hippocampus of rodents was observed [22], and an abnormal location of MAP5 was found in post-mortem AD brains [23 Best known and studied are changes in the tau proteins as key indicators of normal and pathological aging. During AD and other tauopathies, tau aggregates in the somatodendritic compartment of neurons in selected brain regions, where it forms neurofibrillary tangles (NFTs) [24 The redistribution of tau from the axon to the somatodendritic compartment is associated with increased tau phosphorylation [25-28 Tau is also subject to several other post-translational modifications such as O-glycosylation, ubiquitination, nitration, and truncation, all of which can contribute to the pathological changes in tau localization and function [29

It is still controversial whether changes in the organization of the microtubule skeleton play a primary role in the pathological process or are a by-product of degenerating neurons. This is also illustrated by the potential contribution of the tau proteins. The classical view that impairment of the function of tau to interact with microtubules leads to microtubule breakdown in affected neurons has been challenged by the observation that neither chronic nor acute knockdown of tau has an obvious impact on axonal microtubule stability [14, 30 In contrast, a knockdown of tau can even have a protective effect in the case of stress and several neuronal diseases that further question a tau “loss of function” hypothesis [31-33 Thus, disease-related post-translational modifications and the formation of oligomeric tau species can actually induce neurotoxicity, which supports a “gain of toxic function” hypothesis via the involvement of tau in the disease process [34 It is still an open question whether such a “gain of toxic function” is related to a malfunction of microtubules or whether it is a change in the interaction of tau with other cell partners. In fact, the non-microtubule-binding N-terminal region (NTR) of tau has been reported to interact with many proteins that may contribute to its physiological and pathological role [17 Bioinformatic analyses have shown that proteins that have been mapped to specifically interact with the NTR are involved in plasma membrane binding and function, endo-/exocytosis, and various signaling mechanisms [35

Recently, a “tau propagation hypothesis” was developed to explain the stereotypical progression pattern of the formation of NFTs in AD [36 According to this hypothesis, pathological forms of tau can be transmitted between neurons by releasing tau from one neuron and ingesting it into another. In fact, *in vitro* and *in vivo* models showed that pathological forms of extracellular tau are taken up by cells and can induce intracellular tau aggregates [37-39 The propagation properties of tau can depend on its isoforms, its state of aggregation, and post-translational modifications, which can also determine the respective tauopathy [40 The concept remains controversial but has attracted attention as a potential therapeutic target for stopping the progression of tauopathies since reducing extracellular tau could slow the spread of the tau pathology.

**Fig. (1):**
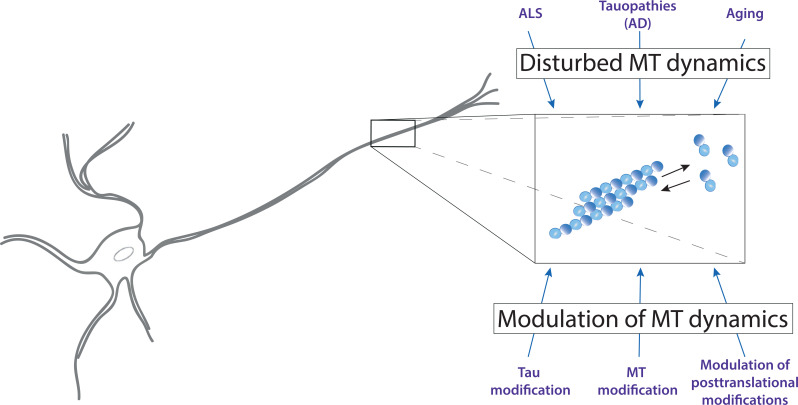
Age- and disease-related disturbance of axonal microtubule polymerization as a potential target of microtubule modulating-agents. Tubulin is shown as blue spheres (light blue: beta tubulin; dark blue: alpha tubulin).

Regardless of whether changes in the microtubular system play a primary role in the disease process, it may still be helpful to stabilize the microtubule skeleton and reduce the potentially toxic activities of intra- or extracellular tau to aid longer survival and to maintain the proper function of the affected neurons in the disease process (Fig. **1**).

## 3 MICROTUBULE-DIRECTED APPROACHES FOR THE TREATMENT OF NEURODEGENERATIVE DISEASES

Several small molecules have been or are in clinical trials aimed at modifying microtubule-associated processes (Table **2**). The drugs target microtubules themselves, target monomeric or aggregated tau protein, or target post-translational modifications as potential modifiers of the microtubule system, such as phosphorylation or O-glycosylation. In addition, several active and passive immunotherapy approaches also target all or are selective for some isoforms of the tau protein (Table **3**). Information of candidate molecules was obtained from ALZForum (https://www.alzforum.org/), ClinicalTrials.gov (https://clinicaltrials.gov/), and AdisInsight – Springer (https://adisinsight.springer.com).

### 3.1 Microtubules as a Drug Target

Modulating microtubule dynamics and correcting impaired microtubule stability can be a useful neuroprotective strategy to counteract the disruption of the microtubule system seen in several neurodegenerative diseases.

A paradigmatic and well characterized molecule is Epothilone D (EpoD, BMS-241027), a small molecule microtubule stabilizer. EpoD is a brain-penetrating synthetic taxol-derived compound [41 In various transgenic tau mouse models, EpoD reduced transport deficits and improved cognition by stabilizing axonal microtubules [42-44 However, dose-dependent neurotoxic effects of EpoD and related drugs have also been identified [45-47 In February 2012, BMS-241027 entered a phase I clinical trial to evaluate its safety, efficacy, and tolerability in patients with mild AD. The clinical study aimed to establish a pharmacodynamic profile for BMS-241027 in cerebrospinal fluid (CSF)-present tau protein. After 9 weeks of intravenous (IV) administration of BMS-241027, safety and cognitive tests using magnetic resonance imaging (MRI) were performed for the patients with mild AD. The study was completed in October 2013. Clinical studies on AD were then discontinued due to a lack of mechanistic selectivity for neurons.

Another small molecule microtubule stabilizer and a synthetic taxane derivative that can cross the blood-brain barrier is TPI-287. TPI-287 has been evaluated for pharmacokinetics, pharmacodynamics, safety, and efficacy in two phase I clinical trials in patients with neurological disorders, mild to moderate AD, corticobasal syndrome, and progressive supranuclear palsy (PSP), respectively. Both studies ended in September 2019. There are currently no active studies on TPI-287.

Davunetide (NAP) is called a “neuronal tubulin-preferring agent” and thereby modulates the pool of microtubules in neurons, but its exact mechanism of action is not clear. Davunetide is derived from a glial-derived growth factor known as activity-dependent neuroprotective protein (ADNP) and is made up of a sequence of eight amino acids with neuroprotective activity. In an AD mouse model, davunetide reduced tau pathology and improved cognitive function [48 It can have pleiotropic functions, which makes it unclear to what extent direct microtubule-related functions are involved in its activity. Davunetide completed two phase I and II clinical trials of PSP. Due to the negative endpoints released after the studies were completed, no further clinical studies were planned.

The disappointing results of clinical trials with general microtubule stabilizers such as EpoD, TPI-287, and perhaps davunetide may also reflect the critical involvement of microtubules in different cell types and biological processes. This makes it problematic to reduce microtubule dynamics in all cell types without distinguishing between neurons and glial cells. In particular, microglia, as the brain’s primary innate immune cells, may need dynamic microtubules to modulate their cell shape and ramification, which are necessary to respond appropriately to tissue injuries or diseases [49

### 3.2 Tau as a Drug Target

As described in paragraph 2, tau proteins are key indicators of pathological aging and tauopathies. Post-translational modifications of tau, such as increased phosphorylation at selected sites or proteolytic cleavage are associated with neurodegenerative diseases. Thus, a reduction in the total amount of tau protein or a selective reduction in disease-associated tau proteoforms could be of therapeutic value.

Several small molecules targeting tau protein have been or are in clinical trials. BIIB080 is an antisense oligonucleotide that inhibits tau mRNA translation, thereby reducing the total amount of tau protein. In preclinical studies, the antisense oligonucleotide showed a decrease in NFT spread and decreased neuronal loss in adult tau-transgenic mice [50 A clinical study to evaluate the safety, tolerability, and pharmacological kinetics and dynamics of BIIB080 in patients with mild AD was initiated in June 2017 and will be completed in May 2022.

Several small molecules such as ACI-3024, TRx0014, TRx0037, and TRx0237 aim to reduce tau aggregation and thereby dissolve or prevent the formation of potentially toxic tau aggregates. It has been claimed that ACI-3024 has the property of selectively disrupting pathological tau aggregates without side effects from other tau species or Aβ plaques. To date, two clinical studies have been conducted for ACI-3024. The first clinical study was phase I and started in July 2019 with the aim of investigating the safety, admissibility, pharmacokinetics, and dynamics of ACI-3024 in healthy volunteers. This study was reported as completed in the AC Immune media release in March 2021. The second study was phase II and started in March 2020 with the aim of determining the drug’s effectiveness as a potential treatment for AD and is ongoing.

TRx0014, TRx0037, and TRx0237 are derivatives of methylthioninium chloride (MTC), also known as methylene blue. They have been reported to inhibit tau aggregation [51 In a preclinical study, methylene blue preserved cognition in a mouse model of tau aggregation [52 TRx0014 and TRx0037 did not show promising results in clinical trials and have been discontinued. TRx0237, a reduced form of methylthioninium chloride, was then developed as a second-generation compound for improved intestinal absorption and, as a result, increased bioavailability. TRx0237 entered phase III clinical trials. It has undergone studies to evaluate its safety and effectiveness in AD patients (mild to moderate cases) and is ongoing with an active study. Another clinical study was also conducted to evaluate the safety and tolerability of TRx0237 in patients with mild to moderate AD who were taking a pre-existing stable acetylcholinesterase inhibitor or memantine therapy. This study was discontinued because of administrative reasons.

The small molecule AZP-2006 is said to block tau phosphorylation by stabilizing the prosaposin-progranulin complex. Progranulin and prosaposin are neuroprotective secreted proteins that correlate with phosphorylated tau levels [53 AZP-2006 was claimed to lower levels of phosphorylated tau and the resulting neuroinflammation in mouse models with the accelerated aging phenotype. A clinical study of AZP-2006 to study the drug's pharmacokinetics in healthy volunteers was conducted in September 2018 and completed in February 2020. A recent phase II clinical trial was conducted in June 2020 in patients with PSP that was expected to end after one year.

Several active and passive immunotherapy studies against various tau proteoforms and tau domains have been completed or are still active. These include active immunizations with a peptide consisting of the mid-domain of tau (AADvac1), the region of tau involved in microtubule binding and tau aggregation, and a tau phosphoepitope (ACI-35). However, most studies used passive immunizations with monoclonal antibodies directed against the middle domain of tau (BIIB076, Bepranemab, E2814), the N-terminal region of tau (Gosuranemab, Semorinemab, Tilavonemab, Zagotenemab), which is believed to aid in tau spreading [54], and potentially neurotoxic phosphorylated tau species (JNJ-63733657, Lu AF87908, PNT001, RG7345). While some of the studies were discontinued due to side effects (RG7345) or failure to meet the endpoint for certain diseases (BIIB076), most of the studies are still ongoing and their effectiveness is not yet known.

The reduction of the tau level can be a promising approach, especially in Alzheimer’s disease, which is associated with increased amounts of tau in neurons and the cerebrospinal fluid [55, 56 This also applies to indications of a toxic gain of function of the tau protein in various neurodegenerative diseases. The physiological role of tau is not fully understood as it does not appear to be required for microtubule stabilization, as discussed in paragraph 2. However, because tau is known to have multiple interacting partners and contribute to various signalling mechanisms, long-term reductions in the total amount of tau in neurons can pose a risk.

In immunotherapeutic approaches, it is important to consider whether the antibodies can penetrate the neurons or only remain in the extracellular space since the tau levels within the cells are considered to be of higher magnitude than in the interstitial and cerebrospinal fluid [57 In fact, some antibodies have been shown to be taken up by neurons [58-60], but this may not be the case for all antibodies as charge and dissociation constants appear to play an important role in cellular uptake [61 Thus, these factors can be an important determinant of effectiveness.

### 3.3 Targeting Post-translational Modifications as Potential Modifiers of the Microtubule System

Several components of the microtubule system undergo post-translational modifications, including tubulin itself, tubulin dimer binding proteins such as stathmin, and MAPs such as tau. Phosphorylation of tau is probably the best studied modification of the microtubule system due to its involvement in tauopathies. Increased phosphorylation of tau at selected sites has been linked to AD, can modulate tau aggregation in NFTs, and affect the localization and function of tau within neurons [62 The phosphorylation of tau at several sites reduces the activity of tau to polymerize microtubules and to bind to other interaction partners such as membrane components [63 Thus, reducing pathological tau phosphorylation, either directly or indirectly, may modulate disease progression.

Tideglusib is a small molecule inhibitor of thiadiazolidinone origin for glycogen synthase kinase 3 beta (GSK-3β), a kinase that phosphorylates tau at multiple positions, including sites that have increased phosphorylation in tau from paired helical filaments (PHFs). Treatment with a thiadiazolidinone compound decreased tau phosphorylation and reduced cell death in a mouse model of AD [64 Tideglusib underwent two clinical studies for AD and one study for PSP. The three studies aimed to evaluate the drug's safety and effectiveness and to determine the effects of its administration on cognitive changes in AD patients. The results were negative as expectations were missed; as a result, studies on Tideglusib in AD and PSP patients were discontinued. However, studies of the drug to treat congenital myotonic dystrophy are still ongoing.

LY3372689 targets another post-translational modification of tau, O-linked N-acetylglucosamine (O-GlcNAc) modification. O-GlcNAcylation is a common post-translational modification of cytoskeletal proteins in which N-acetylglucosamine is added to serine and threonine residues. O-glycosylation is an interesting modification because it blocks potential phosphorylation sites. Therefore, increased O-glycosylation of tau may lead to decreased phosphorylation at selected sites. Consequently, LY3372689 acts as an inhibitor of the enzyme O-GlcNAcase, which removes the glycosyl residue. Treatment with an O-GlcNAcase inhibitor in a mouse model for tauopathies slowed neurodegeneration, reduced tau phosphorylation at disease-relevant sites, and decreased tau aggregation [65, 66 Four clinical studies were performed to examine LY3372689 in healthy subjects. Two studies aimed to investigate the pharmacokinetics, viability, and safety of LY3372689. The other two studies were initiated to assess the O-GlcNAcase occupancy for single and multiple oral doses of LY3372689. All four studies were phase I clinical studies and were conducted between 2019 and 2020.

Several other small molecules may also have a more indirect effect on the phosphorylation state of tau. This is the case with davunetide (NAP) and the various methylene blue derivatives. It has been shown that NAP also inhibits the aggregation of amyloid-β (Aβ) peptide in a mouse model of AD [67] and may thereby indirectly reduce tau phosphorylation by inhibiting Aβ-mediated GSK-3β activation [68 Methylene blue has antioxidant and mitochondrial protective effects [69] and has also been shown to inhibit the potential neurotoxic Aβ oligomerization [70

A general problem with targeting post-translational modifications of proteins of the microtubule system is that the corresponding modifications and enzymes also act on non-microtubule related targets. Phosphorylation is a very general modification that affects the function of various signalling pathways, and individual kinases are also involved in many processes. This is especially true for GSK-3β, which is seen as an important link between Aβ and tau pathology, but dysregulations are also involved in the development of cancer, diabetes, schizophrenia, and bipolar disorder [71 Thus, even very specific targeting of GSK-3β activity can have many undesirable side effects.

The same is true for O-GlcNAcylation. Approximately 1000 proteins have been described as O-GlcNAcylated [72], and like phosphorylation, O-GlcNAcylation can modulate enzymatic activity, protein turnover, protein interactions, and subcellular localization and is involved in a wide variety of biological processes and diseases, including cancer [73

## 4 POSSIBILITIES AND LIMITS WITH REGARD TO THE NEURONAL MICROTUBULE SYSTEM AS A TARGET STRUCTURE FOR DRUG INTERVENTIONS

Microtubules play a crucial role in neuronal stability, and disruption of the microtubule skeleton is a characteristic downstream event in various neurodegenerative diseases. Thus, the stabilization of the microtubule system can have neuroprotective potential and support critical microtubule-dependent processes such as efficient axonal transport.

While the stabilization of microtubules by small molecules, such as EpoD, reduced axonal degeneration in mouse models of tauopathies, no clear beneficial roles have been found in patients so far. A problem with the type of microtubule stabilizers that have so far been in clinical trials is that they act quite non-specifically on microtubules in several cell types and subcellular compartments. While stabilization of microtubules may be beneficial in the axons of neurons affected in tauopathies, similar stabilization of microtubules in dendrites of the same or other neurons can be counterproductive. In fact, even subnanomolar concentrations of EpoD induce a dendritic simplification in organotypic hippocampal slices [74 This suggests that drug-induced hyperstabilization of microtubules in dendrites can negatively affect the connectivity of neurons. Stabilization of microtubules in other cell types in the brain, such as microglia, can also have undesirable side effects. A rearrangement of microtubules is involved in the transition of microglia between a ramified, resting phenotype and an amoeboid, activated state, which is closely related to their function in inflammatory responses of the brain [75 In fact, Epothilone B treatment has been reported to affect functional recovery after spinal cord injury by changing cytokine release, likely released by activated microglia [76

Therefore, tau may be a better target because it is enriched in neurons and pathological changes in tau are associated with several neurodegenerative diseases collectively known as tauopathies. The modulation of the total amount of tau is not likely to affect microtubule stability *per se* since a genetic or functional knockout of tau did not influence microtubule stability. Thus, contrary to popular belief, tau is clearly not a microtubule-stabilizing protein in the axon under physiological conditions (see discussion in paragraph 2). Instead, it has been shown that tau can act as a modifier in various neurological diseases, which indicates a toxic gain of function of the disease-modified tau protein. Current findings support the view that soluble oligomeric tau species act as neurotoxic agents both within cells and, when secreted, on other cells [77-79 Such a neurotoxic tau species probably consists of post-translationally modified tau, in particular tau, which shows increased phosphorylation at specific sites or is proteolytically cleaved at certain positions, which may drive the production of the oligomers [80 However, the physiological function of tau has not yet been clarified. Tau is the product of a gene duplication event at the beginning of vertebrate development, and so far, all vertebrates appear to contain at least one gene encoding tau, suggesting that tau cannot be dispensed with [81 In addition to microtubules, tau has many interaction partners, and in particular, the N-terminal region of tau that does not bind to microtubules can be involved in signal transduction mechanisms or membrane-associated functions [16, 35 Thus, the suppression of tau expression can have undesirable side effects in the long term.

Small molecule interventions or immunization that target potentially toxic proteoforms of tau or tau oligomers may therefore be more advisable. A problem, however, can arise from the observation that the same region involved in tau aggregation is also the region that binds to microtubules. Thus, potential tau aggregation inhibitors that act by binding to the repeat region of tau can also adversely affect the interaction of tau with microtubules and thereby induce undesirable side effects.

## 5 WILL IT EVER WORK? WHAT CAN BE LEARNT FOR FUTURE APPROACHES?

Both general modifiers of microtubule polymerization and stability and drugs that target the general amount of tau act rather non-specifically, and in some cases, it is not even clear whether microtubules or tau are the main targets. This applies in particular to drugs that address post-translational modifications such as GSK-3β inhibitors or inhibitors of O-GlcNAcylation since in addition to components of the microtubule system, many proteins are targets of these enzymes. This also applies to substances such as methylthioninium chloride (MTC), which are claimed to inhibit tau aggregation but whose antioxidant effects can modulate the activity of various enzymes and signalling components. If these substances promote neuron survival, they could still be a useful addition to the toolbox to support overall brain health, but classifying them as microtubule-targeting drugs is misleading.

A lesson from using microtubule stabilizing drugs such as EpoD is that stabilizing microtubules in the axon can be beneficial in tauopathy models, but that microtubules in other compartments require sufficient dynamics to enable structural and functional plasticity. The reduction of microtubule dynamics through drug-induced stabilization of dendritic microtubules leads to a dendritic simplification, which negatively influences the synaptic connectivity, which is certainly counterproductive for efficient communication between neurons. Artificial stabilization of microtubules in other cell types such as microglia or astrocytes can also have undesirable side effects since the microtubule stabilization can negatively influence a dynamic reaction of these cell types to the environment.

One solution could be small molecules that specifically modulate axonal microtubules but are neutral to microtubules in other compartments or cell types. Special features of axonal microtubules are that they are uniformly organized with their plus ends towards the axon tip, are present in fragments, are relatively straight (not curved), have a uniform inter-microtubule distance in the entire axon, and thus have a high concentration of potential binding sites. A cell-permeable small molecule that takes advantage of these properties and specifically binds to a straight, parallel, and dense array of microtubules could be a useful drug to prevent or counteract disease-associated destabilization of axonal microtubules. In fact, MAPs such as doublecortin seem to be able to specifically recognize the curvature of microtubules [82] and tau seems to preferentially bind to sites with high microtubule curvature in cells [83 This suggests that the development of drugs that specifically recognize an axon-like microtubule organization may also be possible. Molecules that mimic the behaviour of the axonally enriched tau proteins by interacting with microtubules through a kiss-and-hop mechanism, thereby modulating microtubule polymerization without disrupting axonal transport, could also form the basis for an axon-specific drug.

With regard to tau as a drug target, it may be advisable to reduce its amount only moderately so as not to impair other tau-dependent interactions and activities. The reduction of potential neurotoxic species such as proteolytically cleaved tau species or post-translationally modified tau at selected residues could be particularly helpful here.

In addition, other components of the axonal microtubule system may be useful drug targets. Under physiological conditions, axonal microtubule stabilization appears to be primarily mediated by MAP6 (STOP protein) [84-86] and drug-induced upregulation of this protein may modulate microtubule stability with a preference for the axon. Reduction of tubulin dimer binding proteins such as stathmins, which shift the equilibrium of microtubule polymerization towards depolymerized microtubules, can in turn be a useful target since a decreased amount of stathmin can, in turn, increase microtubule polymerization and stability. Since stathmin-deficient mice develop age-dependent axonopathy, this can be an interesting drug target [87

## CONCLUSION

Hence, there seems to be some potential to develop new microtubule modulation strategies that could prevent axonal degeneration, but the way things are, we are not there yet.

## Figures and Tables

**Table 1:** Examples of microtubule changes in neurodegenerative diseases.

**Disease Type**	**Changes of Microtubules**	**References**
**Primary Tubulinopathies**		
Hypomyelination with atrophy of the basal ganglia and cerebellum	Patients with mutation in *TUBB4A*, which encodes tubulin bετα-4A	[88]
Congenital-onset spastic paraplegia	Patients with mutation in *TUBB4A*, which encodes tubulin bετα-4A	[89]
Complex cortical malformations	Patients with mutation in *TUBA1A, TUBB2B, TUBB3, TUBB5, TUBG1*	[90]
Changes in post-translational modifications of microtubules		
Charcot-Marie-Tooth disease	Decreased microtubule acetylation in a mouse model of mutant HSPB1-induced Charcot-Marie-Tooth disease	[91]
Alzheimer’s disease	Reduction of acetylated alpha-tubulin immunoreactivity in neurofibrillary tangle-bearing neurons in Alzheimer’s disease patients	[92]
Huntington disease	Reduced levels of acetylated tubulin in the brains of patients affected by Huntington disease	[93]
Alzheimer’s disease	Glutamylation on tubulin is increased in the hippocampi of patients with Alzheimer’s disease	[94]
Alzheimer’s disease	Polyglutamylation, tyrosination, and detyrosination are proportionally reduced in patients with Alzheimer’s disease. Increase in the proportion of the acetylated α-tubulin in the remaining α-tubulin (overall decrease of α-tubulin)	[95]
Microcephaly, intellectual disability and delayed gross motor and speech development	Reduction of detyrosinated tubulin in brain tissue	[96]
Changes induced by microtubule modulating proteins		
All tau related diseases	Site-specific pseudophosphorylation of tau promotes distinct microtubule organizations: stable single microtubules, stable bundles, or dynamic bundles. Disease-related tau mutations increase the formation of highly dynamic bundles.	[97]
Alzheimer Disease and Hereditary Spastic Paraplegia	Loss of microtubules due to polyglutamylation of microtubules, which acts as a trigger for spastin mediated severing of dendritic microtubules in AD. Spastin function in terms of microtubule severing is decreased at least for the gene product of the mutated allele, resulting in overstable microtubules in disease model systems of HSP	[98]
Parkinson’s disease	Parkin binds to alpha/beta tubulin and increases their ubiquitination and degradation	[99]
Parkinson’s disease	Leucine-rich repeat kinase 2 regulates tubulin acetylation	[100]

**Table 2:** Clinical trials of tau or microtubule-targeting small molecules against neurodegenerative diseases.

**Drug**	**Synonym**	**Assumed Mechanism of Action**	**Class**	**Originator/Developer**	**Type**	**Clinical Trials Regarding ** **Neurodegenerative Diseases**	**FDA Clinical Trials**	**Comment**
**ACI-3024**	Tau Morphomer^TM^	Target: Tau protein. Inhibits tau aggregation, disaggregates neurofibrillary tangles.	Anti-tauopathy	AC Immune SA Country: Switzerland	Small molecule	First-in-human, randomized, placebo-controlled, double-blind, sequential single and multiple ascending dose (SAD/MAD) study with open-label food effect and pharmacodynamic assessment arms to assess the safety, tolerability, pharmacokinetics, and pharmacodynamics of ACI-3024 Completed: March 2021. Phase II clinical trial of a tau morphomer (ACI-3024) for the treatment of AD. Status: Still ongoing.	Phase I/II	Ongoing
**AZP-2006**	Ezeprogind - AlzProtect	Target: Tau protein. Blocks tau phosphorylation by stabilizing the prosaposin-progranulin complex.	Antidementias, Neuroprotectants, Anti tau-hyperphosphorylation	AlzProtect Country: France	Small molecule	A study assessing the potential effects of food intake on pharmacokinetics of AZP2006 in healthy volunteers. Completed: February 20^th^, 2019. Multi-center, randomized, double-blind, placebo-controlled, parallel group study to assess tolerability, safety, pharmacokinetics and effect of AZP2006 on cerebrospinal fluid biomarkers in 36 patients with Progressive Supranuclear Palsy (PSP). Estimated completion date: June 30^th^, 2021.	Phase II	Ongoing and recruiting
**BIIB080**	ISIS 814907, IONIS BIIB4_RX_, IONIS-MAPTR_X_	Target: Tau protein. Interferes with tau expression by inhibiting tau mRNAs translation into protein.	Tau mRNA translation inhibitor	IONIS Pharmaceuticals, Inc., Biogen Country: US/US	Antisense oligonucleotide	Randomized, double-blind, placebo-controlled study, followed by an open-label extension, to evaluate the safety, tolerability, pharmacokinetics and pharmacodynamics of multiple ascending doses of intrathecally administered ISIS 814907 in patients with mild AD. Estimated Completion: May 2022.	Phase I/II	Active
**BMS-241027**	Epothilone D	Target: Tau protein; tubulin; microtubules. Tau protein inhibitor; Tubulin modulator; Microtubule stabilizer.	Antidementia, microtubule stabilizer	Bristol-Myers Squibb Country: USA	Small molecule	Randomized, double-blind, placebo-controlled, multiple ascending dose study to evaluate the safety, tolerability and pharmacokinetics of a microtubule stabilizer (BMS-241027) in healthy females. Completed: July 2013. Phase I, multi-center, randomized, double-blind, placebo-controlled study to evaluate the safety, tolerability and the effect of BMS-241027 on cerebrospinal fluid biomarkers in subjects with mild AD. Completed: October 2013.	Discontinued	Lacks neuronal selectivity
**Davunetide**	NAP, AL-108	Target: Microtubules MT stabilizer and decreases tau phosphorylation	Antidementia	Allon Therapeutics Inc. Country: Canada	Neuropeptide	12 week randomized, double blind, placebo-controlled pilot study of Davunetide (NAP, AL-108) in predicted tauopathies. Completed: December 2010. Phase II/III, randomized, double-blind, placebo-controlled, study to evaluate the safety and efficacy of Davunetide for the treatment of Progressive Supranuclear Palsy. Completed: December 2012	Phase II	Stopped for PSP (negative endpoints)
**LY3372689**	O-GlcNAcase inhibitor - Eli Lilly and Company	Target: Tau protein/O-GlcNAcase. Inhibits O-GlcNAcase that in turn reduces tau aggregation.	Anti-tauopathy, Antidementia	Eli Lilly and Company Country: USA	Small molecule	Single-ascending dose, safety, tolerability, and pharmacokinetic study of LY3372689. Completed: June 24^th^, 2019. Multiple-ascending dose, safety, tolerability, and pharmacokinetic study of LY3372689. Completed: February 12^th^, 2020. Assessment of brain O-GlcNAcase (OGA) enzyme occupancy after single oral doses of LY3372689 as measured by positron emission tomography (PET) with the radioligand [18F]LSN3316612 in healthy subjects. Completed: February 19^th^, 2020 Assessment of brain O-GlcNAcase (OGA) enzyme occupancy after multiple oral doses of LY3372689 as measured by positron emission tomography (PET) with the radioligand [18F]LY3316612 in healthy subjects. Completed: October 14^th^, 2020.	Phase I	Completed
**Tideglusib**	NP-12, AMO-02 NP031112, Nypta®, Zentylor™	Target: GSK3-β. Inhibits GSK3-β that in turn decreases tau hyperphosphorylation	GSK3-β inhibitor	Zeltia Group/AMO Pharma Country: Spain/US	Small molecule	Phase IIa 20 week double-blind, placebo-controlled, randomized, escalating dose study to evaluate the safety and tolerability of four oral doses of NP031112, a novel GSK3 Inhibitor, in mild to moderate AD patients with stable anticholinesterasic treatment. Completed: November 2009. A double-blind, placebo-controlled, randomized, parallel-group study evaluating the safety, tolerability, and efficacy of two different oral doses of NP031112, a GSK-3 inhibitor, versus placebo in the treatment of patients with mild-to-moderate Progressive Supranuclear Palsy (PSP). Completed: November 2011. Multicenter, randomized, double-blind, placebo-controlled, 4-arm, 26 week parallel-group study to evaluate efficacy, safety and tolerability of 2 oral doses and 2 regimes of Tideglusib vs placebo in mild-to-moderate AD patients. Completed: October 2012.	Phase II	Discontinued for AD and PSP. Ongoing for congenital Myotonic dystrophy
**TPI-287**	ARC-100; NBT-287	Target: Tubulin and microtubules. Microtubule stabilizing agent and Tubulin binder.	Taxanes Antidementias, Antineoplastics	Tapestry Pharmaceuticals, Inc.; Cortice Biosciences Country: US/US	Small molecule	Phase I, randomized, double-blind, placebo-controlled, sequential cohort, dose-ranging study of the safety, tolerability, pharmacokinetics, pharmacodynamics, and preliminary efficacy of TPI 287 in patients with primary four-repeat tauopathies: Corticobasal Syndrome or Progressive Supranuclear Palsy (PSP). Completed: September 2019. Phase I, randomized, double-blind, placebo-controlled, sequential cohort, dose-ranging study of the safety, tolerability, pharmacokinetics, pharmacodynamics, and preliminary efficacy of TPI-287 in patients with mild to moderate AD. Completed: September 2019.	Phase I	Inactive
**TRx0014**	RemberTM, Methylene blue - TauRx	Target: Tau protein/ aggregates. Prevents/dissolves tau aggregates in tauopathies.	Tau aggregation inhibitor	TauRx Therapeutics, Ltd. Country: Singapore	Small molecule	An exploratory placebo-controlled, dose-ranging study of the effects of TRx0014 30 mg TID, 60 mg TID and 100 mg TID in patients with mild or moderate dementia of the Alzheimer type. Completed: December 2007. Comparative bioavailability of TRx0037 and TRx0014 in healthy elderly volunteers. Completed: April 2010. Open label continuation study of the effects of TRx0014 30 mg TID and 60 mg TID in patients with AD. Completed: December 2^nd^, 2010.	Phase II	Discontinued
**TRx0037**	-	Target: Tau protein. Inhibits tau aggregates	Tau aggregation inhibitor	TauRx Therapeutics, Ltd. Country: Singapore	Small molecule	Comparative bioavailability of TRx0037 and TRx0014 in healthy elderly volunteers. Completed: April 2010. Phase I study consisting of a double-blind, placebo controlled multiple dose study of TRx0037 in healthy elderly volunteers. Completed: May 2010.	Phase I	Discontinued
**TRx0237**	LMTM, LMT-X, Leuco-methylthioninium-bis(Hydromethanesulfonate), Hydromethylthionine mesylate - TauRx Pharmaceuticals	Target: Tau protein/ aggregates. Prevent/dissolve tau aggregates in tauopathies.	Tau aggregation inhibitor	TauRx Therapeutics, Ltd. Country: Singapore	Small molecule	Double-blind, placebo-controlled, randomised, 4-week safety and tolerability study of TRx0237 in subjects with mild to moderate AD on pre-existing stable acetylcholinesterase inhibitor and/or Memantine therapy. Started in September 2012, was terminated for administrative reasons. Randomized, double-blind, placebo-controlled, parallel-group, 15-month trial of TRx0237 in subjects with mild to moderate AD. Completed: November 2015. Randomized, double-blind, placebo-controlled, parallel-group, 18-month safety and efficacy study of TRx0237 in subjects with mild AD. Completed: May 2016. Randomized, double-blind, placebo-controlled, three-arm, 12-month, safety and efficacy study of TRx0237 monotherapy in subjects with AD followed by a 12-month open-label treatment. Estimated completion date: June 2023.	Phase III	Active

**Table 3:** Clinical trials of active or passive immunotherapy targeting tau.

**Drug**	**Synonym**	**Assumed Mechanism of Action**	**Class**	**Originator/Developer**	**Type**	**Clinical Trials Regarding ** **Neurodegenerative Diseases**	**FDA Clinical Trials**	**Comment**
**AADvac1**	Axon peptide 108 conjugated to KLH	Target: Tau protein. Evokes immune response against tauopathies.	Immuno-therapy (active)	Axon Neuroscience SE Country: Slovakia	Synthetic peptide	3-months randomized, placebo-controlled, parallel group, double-blinded, multi-center, phase I study to assess tolerability and safety of AADvac1 applied to patients with mild-moderate AD with 3-months open label extension. Completed: March 2015. 18-months open label phase I follow-up study on patients with AD who have completed the AADvac1 phase I study “AXON CO 18700”. Completed: December 2016. 24 months randomised, placebo-controlled, parallel group, double blinded, multi center, phase II study to assess safety and efficacy of AADvac1 applied to patients with mild AD. Completed: June 2019.	Phase II	Active, and under investiga-tions
**ACI-35**	-	Target: Tau protein. Immune response (neither including B cells immune nor T cells); targeted against specific forms of pathological phosphorylated tau.	Immuno-therapy (active)	AC Immune SA, Janssen Pharma-ceuticals Country: Switzerland/Belgium	Synthetic tau fragment	Phase I double-blind, randomized, placebo-controlled study of the safety, tolerability and immunogenicity of ACI-35 in patients with mild to moderate AD. Completed: July 3^rd^, 2017 Phase Ib/IIa multicenter, double-blind, randomized, placebo-controlled study to evaluate the safety, tolerability and immunogenicity of different doses, regimens and combinations of tau targeted vaccines in subjects with early AD. Estimated completion date: Oct. 31st, 2023	Phase II	Active and recruiting
**BIIB076**	NI-105, 6C5 (previous name)	Target: Tau protein. Targeted against the mid-domain of tau to decrease the spread of pathological tau aggregates across neurons.	Immuno-therapy (passive)	Neurimmune AG/Biogen Country: Switzerland/USA	Mono-clonal antibody	Phase I, randomized, blinded, placebo-controlled, single-ascending-dose study to evaluate the safety, tolerability, and pharmacokinetics of BIIB076 in healthy volunteers and subjects with AD. Completed: March 3^rd^, 2020.	Phase I	Still under investi-gation
**Bepranemab**	UCB-0107, Antibody D	Target: Tau protein. Anti-tau antibody that acts on the mid-tau region to limit tauopathies propagation.	Immuno-therapy (passive)	UCB S.A., Hoffmann-La Roche Country: Belgium/Switzerland	Mono-clonal antibody	Subject-blind, Investigator-blind, randomized, placebo-controlled, first-in-human study to evaluate safety and tolerability, pharmacokinetics, and pharmacodynamics of single ascending intravenous doses of UCB0107 in healthy male subjects. Completed: December 1^st^, 2018. Single-center, investigator-blind, subject blind, randomized, placebo-controlled study to evaluate safety and tolerability and pharmacokinetics of single doses of UCB0107 in healthy Japanese subjects. Completed: March 11^th^, 2019	Phase I	Active for PSP study. Planning a proof of concept study for AD
						Participant-blind, investigator-blind, placebo-controlled, phase Ib study to evaluate the safety, tolerability, and pharmacokinetics of UCB0107 in study participants with Progressive Supranuclear Palsy (PSP). Estimated completion date: April 1^st^, 2022 Open-label extension study to evaluate the safety and tolerability of long-term UCB0107 administration in study participants with Progressive Supranuclear Palsy (PSP). Estimated completion date: November 2026		
**E2814**	-	Target: Tau protein. Anti-tau antibody that targets the HVPGG epitope found in tauopathies.	Immuno-therapy (passive)	Eisai Co., Ltd. Country: Japan	Mono-clonal antibody	Randomized, double-blind, placebo-controlled, single ascending dose study to assess safety, tolerability, pharmacokinetics, immunogenicity, and pharmacodynamics of intravenous infusions of E2814 in healthy subjects Estimated completion date: August 27^th^, 2021	Phase I	Active, not recruiting. Planning study on AD patient (not yet recruiting)
**Gosuranemab**	BIIB092, BMS-986168, IPN007	Target: Tau protein. Anti-tau antibody that targets the N-terminal fragments of tau pathological forms to limit its propagation.	Immuno-therapy (passive)	iPierian/Biogen, Bristol-Myers Squibb Country: USA/USA	Mono-clonal antibody	Randomized, double-blind, placebo-controlled, single ascending dose study of intravenously administered BMS-986168 in healthy subjects. Completed: April 30^th^, 2016 Phase Ib, randomized, double-blind, placebo-controlled, parallel cohort safety, tolerability, pharmacokinetics, pharmacodynamics and preliminary efficacy study of intravenously infused BIIB092 in patients with four different primary tauopathy syndromes. Completed: December 13^th^, 2019 Randomized, double-blind, placebo-controlled, parallel-group study to assess the safety, tolerability, and efficacy of BIIB092 in subjects with mild cognitive impairment due to AD or with mild AD. Planned completion date: March 26^th^, 2024 Randomized, double-blind, placebo-controlled, multiple ascending dose study of intravenously administered BMS-986168 in patients with Progressive Supranuclear Palsy (PSP). Completed: October 19^th^, 2016 Randomized, double-blind, placebo-controlled, parallel-group study to evaluate the efficacy and safety of intravenously administered BIIB092 in participants with Progressive Supranuclear Palsy (PSP). Completed: February 7^th^, 2020. Multicenter, open-label, long-term treatment study of intravenously administered BIIB092 in patients with Progressive Supranuclear Palsy (PSP) who participated in Study CN002003. Completed: March 31^st^, 2020	Phase II	Active for AD. Discontinued for PSP because of missing the endpoint of desire
**JNJ-63733657**	JNJ-3657	Target: Tau protein. Anti-phospho-tau antibody that recognizes residue 217 in the pathogenic phosphorylated tau forms.	Immuno-therapy (passive)	Janssen Research & Development, LLC Country: Belgium	Mono-clonal antibody	Randomized, placebo-controlled, double-blind, single ascending dose study to investigate safety, tolerability, pharmacokinetics and pharmacodynamics of JNJ-63733657 in healthy Japanese Ssubjects. Completed: July 11^th^, 2019 2-part randomized, placebo-controlled, double-blind, single and multiple ascending dose study to investigate safety and tolerability, pharmacokinetics and pharmacodynamics of JNJ-63733657 in healthy subjects and subjects with AD. Completed: December 16^th^, 2019 Randomized, double-blind, placebo-controlled, parallel-group, multicenter study to assess the efficacy and safety of JNJ-63733657 in participants with early AD. Completed: March 7^th^, 2025.	Phase II	Targets early AD patient. Active, and recruiting
**Lu AF87908**	-	Target: Tau protein. Slows down the propagation of pathological tau aggregates, and mediates their lysosomal degradation.	Immuno-therapy (passive)	H. Lundbeck A/S Country: Denmark	Mono-clonal antibody	Interventional, randomized, double-blind, placebo-controlled, single-ascending-dose study investigating the safety, tolerability, and pharmacokinetic properties of Lu AF87908 in healthy subjects and patients with AD. Estimated completion date: May 2021.	Phase I	Targets AD, currently active and recruiting
**PNT001**	-	Target: Tau protein. Binds to the cis conformation of phospho-tau at Thr231 to decrease toxic aggregates.	Immuno-therapy (passive)	Pinteon Therapeutics, Inc. Country: USA	Mono-clonal antibody	Phase I, randomized, double-blind, placebo-controlled, single-ascending-dose trial to evaluate the safety, tolerability, immunogenicity, and pharmacokinetics of intravenous PNT001 in healthy volunteers. Completed: February 15^th^, 2021	Phase I	Completed phase I study – Active for AD and traumatic brain injury
**RG7345**	RO6926496	Target: Tau protein. Targets phospho-tau at S422 to reduce tau aggregation.	Immuno-therapy (passive)	Hoffmann-La Roche Country: Switzerland	Mono-clonal antibody	A single-center, randomized, investigator/subject blind, single ascending dose, placebo controlled, parallel study to investigate the safety, tolerability and pharmacokinetics of RO6926496 following intravenous infusion in healthy subjects. Completed: October 1^st^, 2015	Discontinued	Terminated due to incidence of adverse events
**Semorinemab**	RO7105705, MTAU-9937A, RG6100	Target: Tau protein. Binds to the N-terminus of all six CNS tau isoforms.	Immuno-therapy (passive)	AC Immune SA/Genentech (part of Hoffmann-La Roche) Country: Switzerland/USA	Anti-Tau IgG4 antibody	Phase I, randomized, placebo-controlled, double-blind, single and multiple ascending dose study to assess the safety, tolerability, and pharmacokinetics of intravenous and subcutaneous RO7105705 administered in healthy volunteers and patients with mild-to-moderate AD. Completed: June 26^th^, 2017 Phase II, multicenter, randomized, double-blind, placebo-controlled, parallel-group, efficacy, and safety study of MTAU9937A in patients with prodromal to mild AD. Completed: January 15^th^, 2021 Phase II, multicenter, randomized, double-blind, placebo-controlled, parallel-group, efficacy, and safety study of MTAU9937A in patients with moderate AD. Completed: June 3^rd^, 2023	Phase II	Active for AD research
**Tilavonemab**	ABBV-8E12, C_2_N 8E12	Target: Tau protein. Anti-tau antibody that targets the N-terminal fragments of tauopathies targeting the progression of NFTs.	Immuno-therapy (passive)	AbbVie, C_2_N Diagnostics, LLC Country: USA	Humanized IgG4 antibody	Double-blind, placebo controlled, single ascending dose study to assess the safety, tolerability, and pharmacokinetics of C_2_N-8E12 in subjects with Progressive Supranuclear Palsy (PSP). Completed: August 2016. Randomized, double-blind, placebo-controlled multiple dose study to assess efficacy, safety, tolerability, and pharmacokinetics of ABBV-8E12 in Progressive Supranuclear Palsy (PSP). Completed: November 20^th^, 2019 Extension Study of ABBV-8E12 in patients with progressive Supranuclear Palsy (PSP) who completed study C_2_N-8E12-WW-104. Completed: November 20^th^, 2019 Extension Study of ABBV-8E12 in Progressive Supranuclear Palsy (PSP). Completed: December 13^th^, 2019 Phase II multiple dose, multicenter, randomized, double-blind, placebo-controlled study to evaluate the efficacy and safety of ABBV-8E12 in subjects with early AD. Estimated completion date: July 23^rd^, 2021 Extension Study of ABBV-8E12 in Early AD. Estimated completion date: July 14^th^, 2026	Phase II	PSP; Discontinued due to low efficacy in AD; ongoing in Phase II
**Zagotenemab**	LY3303560	Target: Tau protein. Recognizes tau’s N-terminus, targeting soluble tau pathological accumulations.	Immuno-therapy (passive)	Eli Lilly & Co. Country: USA	Humanized anti-Tau IgG4 antibody	Single-dose, dose-escalation study with LY3303560 to evaluate the safety, tolerability, and pharmacokinetics in healthy subjects and patients with mild cognitive impairment due to AD or mild to moderate AD. Completed: July 10^th^, 2018 Multiple-dose, dose-escalation study to assess the safety, tolerability, pharmacokinetics and pharmacodynamics of LY3303560 in patients with mild cognitive impairment due to AD or mild to moderate AD. Completed: June 5^th^, 2019 Assessment of safety, tolerability, and efficacy of LY3303560 in early symptomatic AD. Estimated Completion date: October 22^nd^, 2021	Phase II	Active for AD
